# Supplementary material for: Generation of orthotopically functional salivary gland from embryonic stem cells
Source: Nat Commun. 2018 Oct 11;9:4216. doi: 10.1038/s41467-018-06469-7 (PMC6181987; doi:10.1038/s41467-018-06469-7)
Supplement: Supplementary file 1 — Supplementary Information [file 41467_2018_6469_MOESM1_ESM.pdf]

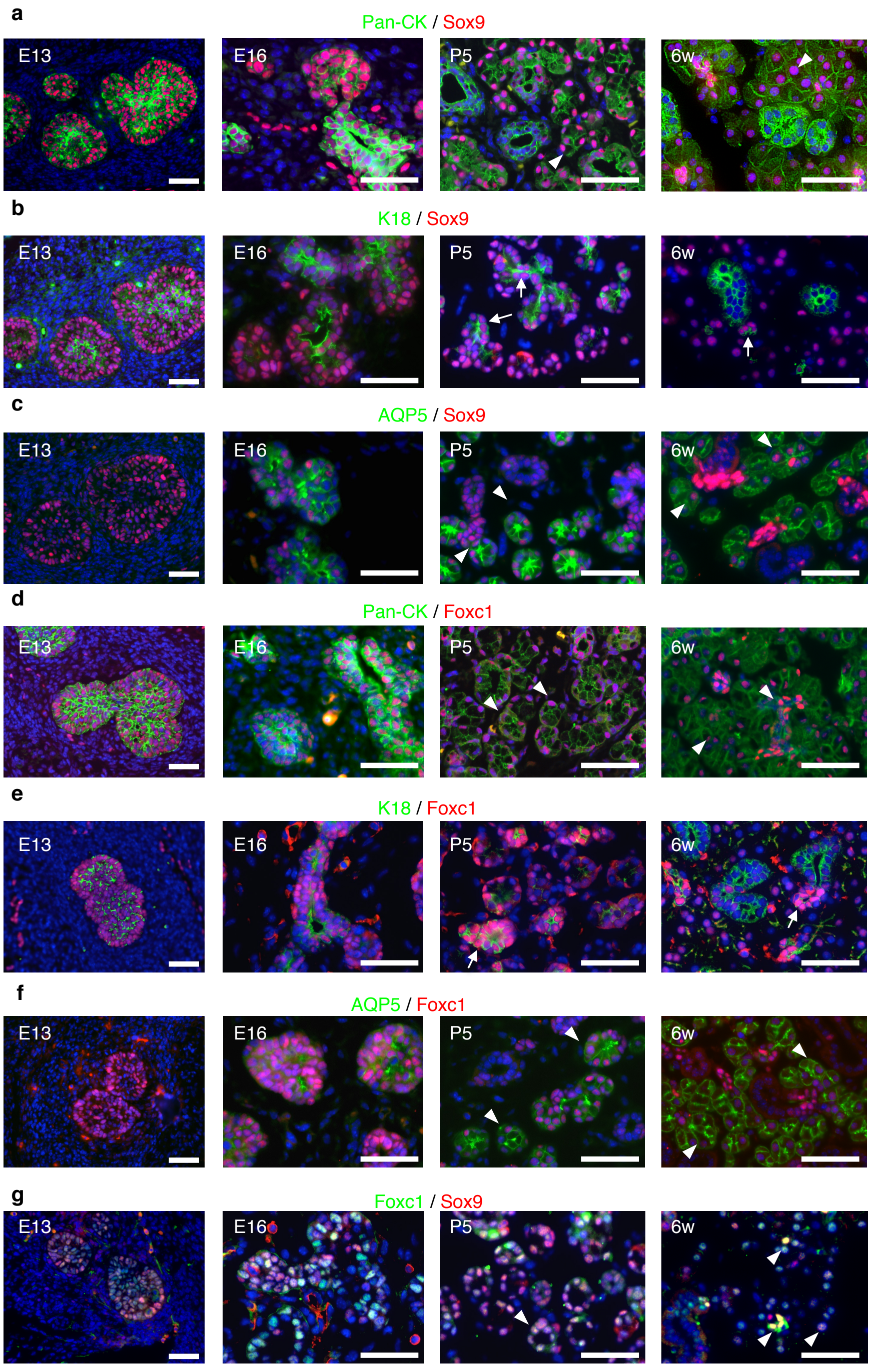

Supplementary Figure 1. Protein expression of Sox9 and Foxc1 during mouse submandibular gland organogenesis. **a** Two embryonic stages (E13.5 and E16.5) and two postnatal stages (P5 and 6w) of SMG were analysed for Sox9 expression. Immunofluorescence staining of Sox9 (red) and Pan-CK (marker of epithelial cells) (green) was conducted during SMG development. At embryonic stages, Sox9 was expressed in the nuclei of the most parenchymal epithelial cells. At postnatal stages, Pan-CK-positive epithelial cells partly expressed Sox9 (arrowhead). **b** Immunofluorescence staining of Sox9 (red) and K18 (marker of ductal cells) (green) was conducted. At embryonic stages, Sox9 was expressed in the nuclei of the most K18-positive cells. At postnatal stages, Sox9-positive cells were localized in K18-positive intercalated ductal cells at 6 weeks (arrow). **c** Immunofluorescence staining of Sox9 (red) and AQP5 (marker of acinar cells) (green) was conducted. At embryonic stages, Sox9 was expressed in the nuclei of the most AQP5-positive cells. At postnatal stages, most acinar cells expressed Sox9 (arrow). **d** Immunofluorescence staining of Foxc1 (red) and Pan-CK (marker of epithelial cells) (green) was conducted during SMG development. At embryonic stages, Foxc1 was expressed in the nuclei of the most parenchymal epithelial cells. At postnatal stages, Pan-CK-positive epithelial cells partly expressed Foxc1 (arrowhead). **e** Immunofluorescence staining of Foxc1 (red) and K18 (marker of ductal cells) (green) was conducted. At embryonic stages, Foxc1 was expressed in the nuclei of the most K18-positive cells. At postnatal stages, Foxc1-positive cells were localized in K18-positive intercalated ductal cells at 6 weeks (arrow). **f** Immunofluorescence staining of Foxc1 (red) and AQP5 (marker of acinar cells) (green) was conducted. At embryonic stages, Foxc1 was expressed in the nuclei of the most AQP5-positive cells. At postnatal stages, most acinar cells expressed Foxc1 (arrow). **g** Immunofluorescence staining of Sox9 (red) and Foxc1 (green) was conducted. Sox9-positive cells was mostly overlapped with Foxc1-positive cells at E13.5, E16.5, P5, and 6w (arrow). Representative images from one out of more than three experiments are shown. Scale bars, 50 μm.

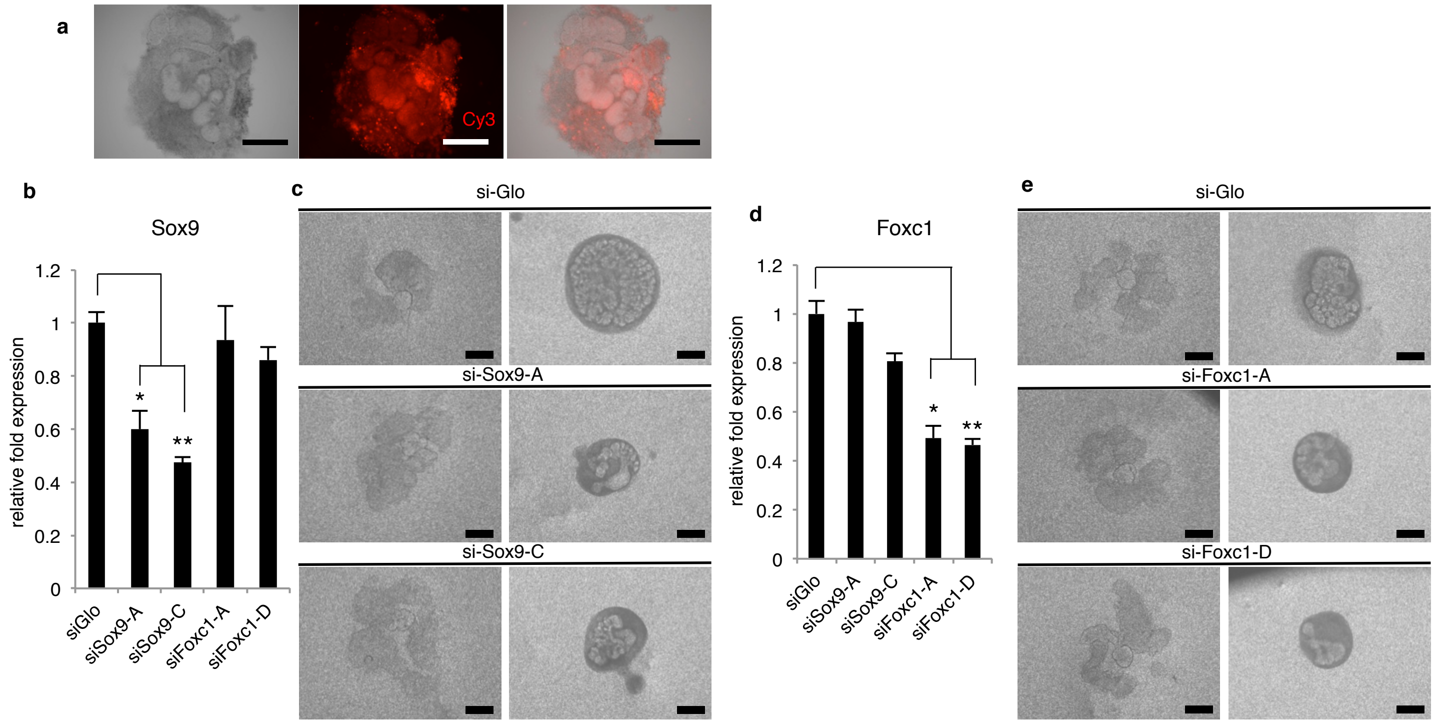

Supplementary Figure 2. Role of Sox9 in the embryonic salivary gland. **a** Epithelial rudiment with mesenchyme was isolated from E13.5 mice and epithelial rudiment was separated from surrounding mesenchyme. Subsequently, epithelial rudiment was recombined with mesenchyme in the presence of siRNA to enhance transfection efficiency of siRNA. Cy3-labelled 500 nM control siRNA (si-Glo) was transfected into recombined SMGs. The recombined SMGs cultured *ex vivo* for 1 day. Brightfield (left), Cy-3 fluorescent (middle), and overlaid (right) images of recombined SMGs were shown. Representative images from one out of more than 6 glands are shown. **b** Sox9 expression levels in an organ-cultured SMG was evaluated via qRT-PCR on day 3. *P = 0.0013, **P = 0.00017. This experiment was replicated three times with similar results. **c** Salivary gland branching was inhibited via knockdown of Sox9 using two Sox9-specifc siRNA (si-Sox9-A and si-Sox9-C) (middle and bottom) compared with the control siRNA (si-Glo) (top). Representative images from one out of more than 10 glands are shown. **d** Foxc1 expression levels in an organ-cultured SMG was evaluated via qRT-PCR on day 3. *P = 0.00013, **P = 0.0004. This experiment was replicated three times with similar results. **e** Salivary gland branching was inhibited via knockdown of Foxc1 using two siRNA (si-Foxc1-A and si-Foxc1-D) (middle and bottom) compared with the control siRNA (si-Glo) (top). Representative images from one out of more than 10 glands are shown. Scale bars: 300 μm. The results are presented as the mean ± S.D. and were normalized to GAPDH. Statistical analyses were performed using Student's t-test.

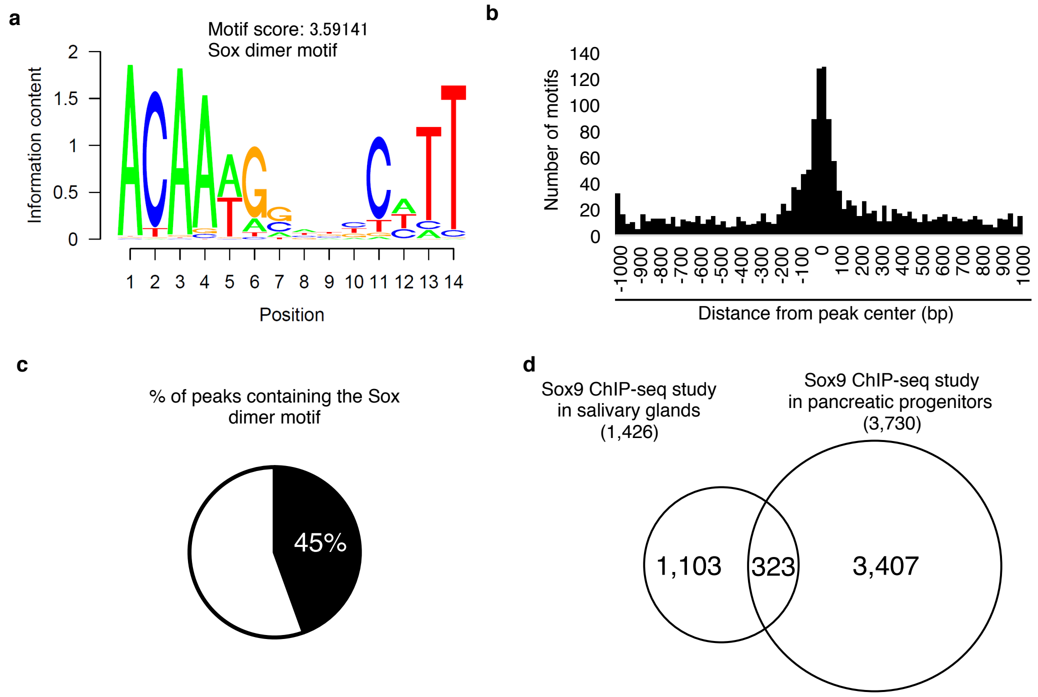

Supplementary Figure 3. Summary of de novo motif analysis on Sox9 ChIP-seq peaks in salivary glands using Cisgenome tool. **a** The top enriched motif logo and the motif score. Predicted motif was Sox dimer motif. **b** The distribution of the motif within a defined interval around each peak center (+/- 1000 bp). **c** The percentage of peaks that contain the motif. **d** Intersection of genes associated with Sox9 ChIP-seq peaks in salivary glands and those in pancreatic progenitors. Sox9-associated 1,426 genes in salivary glands were compared with Sox9-associated 3,730 genes in pancreatic progenitors. The obtained genes in the intersection was listed in Supplementary Data 3.

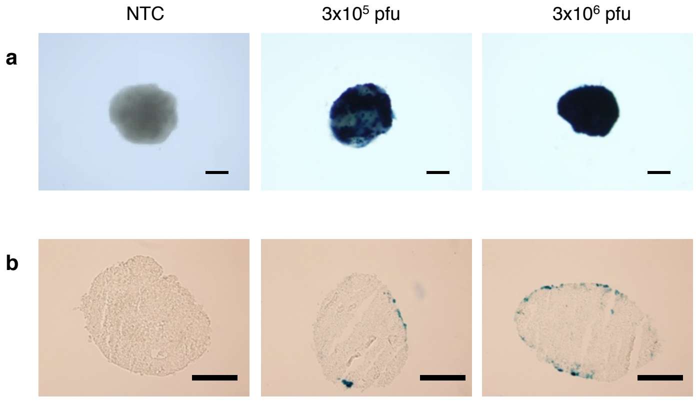

Supplementary Figure 4. Most outer layer cells in ESC aggregates show β-galactosidase activity after infection with a recombinant adenovirus encoding β-galactosidase. **a** Aggregates were infected with a recombinant adenovirus encoding E. coli β-galactosidase at 3×10^5 or 3×10^6 pfu in 100 μl of culture medium. The representative photographs demonstrate that most of the outer cells in the aggregate showed β-galactosidase activity based on X-gal staining. **b** Sections of the aggregates were also stained with X-gal (blue) 24 hours after infection. X-gal-positive cells can be observed in the outer layer of the aggregates. Representative images from one out of more than three experiments are shown. Scale bars, 300 μm.

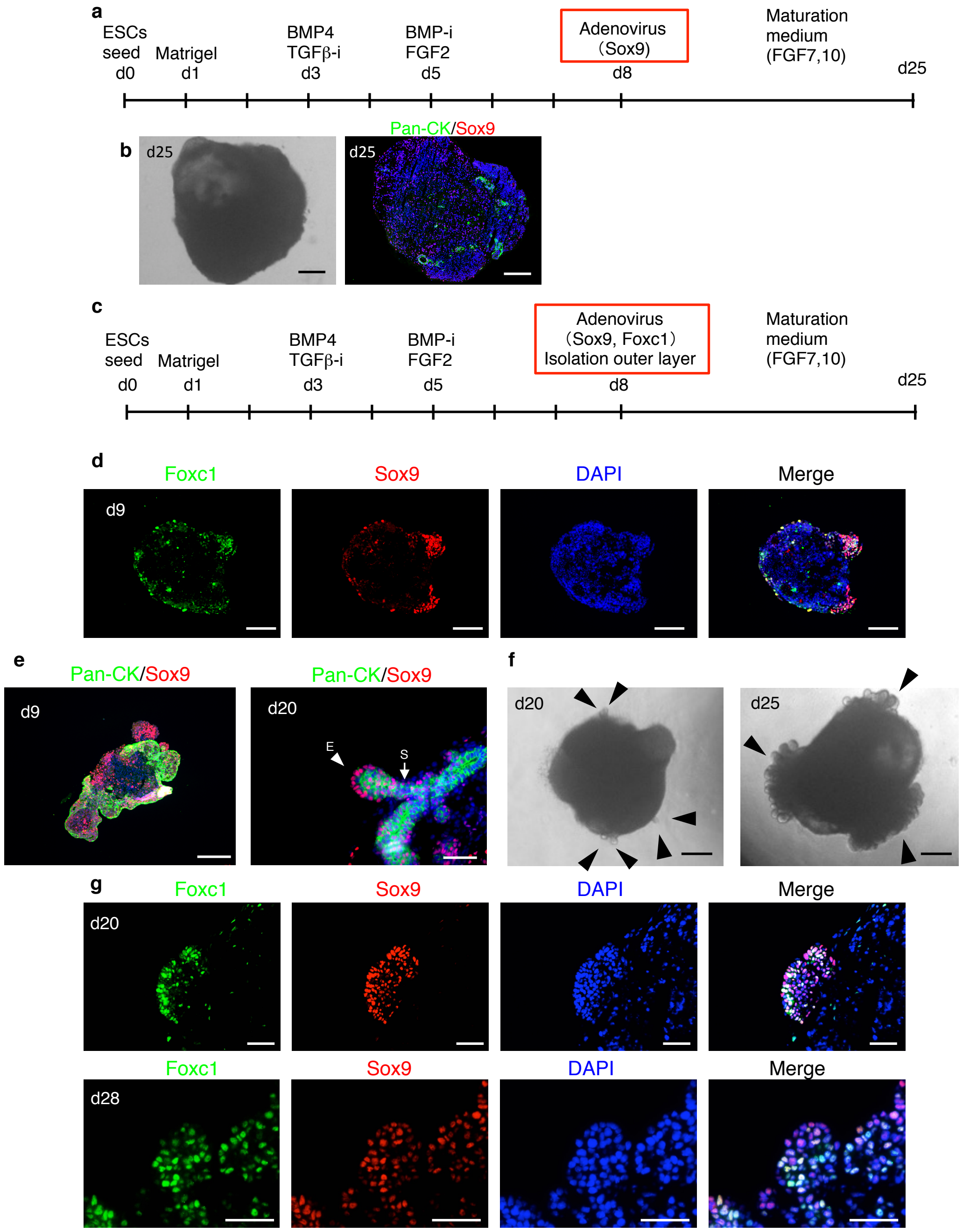

Supplementary Figure 5. Overexpression of Sox9 and Foxc1 gene induces ESC-derived oral epithelium differentiation into salivary gland rudiment. **a** Protocol for ESC aggregate culture. Adenoviral infection was conducted on d8 of differentiation. The aggregates were cultured until d25 in the presence of FGF7 and 10. **b** Representative phase-contrast image of aggregate on d25 of differentiation (left). The aggregates were enlarged, but no remarkable morphological changes were observed. Immunofluorescence staining of Pan-CK (green) and Sox9 (red) in the infected aggregates on d25 of differentiation (right). Both Pan-CK and Sox9-positive cells nearly disappeared. Scale bars, 200 μm. This experiment was replicated three times with similar results. **c** Protocol for ESC aggregate culture. Adenoviral infection (Sox9, Foxc1) was conducted on d8 of differentiation. The outer layer of the aggregates was isolated and cultured until d25 in the presence of FGF7 and 10. **d** Immunofluorescence staining of Foxc1 (green) and Sox9 (red) on d9. Foxc1 and Sox9 double-positive cells detected in the outer layer cells of the adenovirus-infected aggregates. This experiment was replicated three times with similar results. Scale bars, 100 μm **e** Immunofluorescence staining of Pan-CK (green) and Sox9 (red) on d9 (left) and d20 (right). Isolated outer layer contained Sox9 expressing epithelial cells (left). The epithelial bud on d20 showed positivity for Sox9 and the structure was similar to the initial bud stage of the salivary gland. E; epithelial bud, S; stalk. Scale bars, 300 μm (left) and 50 μm (right). **f** Representative phase-contrast images of the aggregates on d20 (left) and d25 (right). Arrowheads indicate epithelial buds protruding from the outer layer. Scale bars, 200 μm. **g** Immunofluorescence staining of Foxc1 (green) and Sox9 (red) on d20 and d28. Foxc1 and Sox9 double positive cell clusters were detected in outer layer of aggregate on d20(top). The bud structures on d28 consisted of Foxc1 and Sox9 double-positive cells. Representative images from one out of more than three experiments are shown. This experiment was replicated three times with similar results. The nucleus can be seen in blue (DAPI). Scale bars, 50 μm.

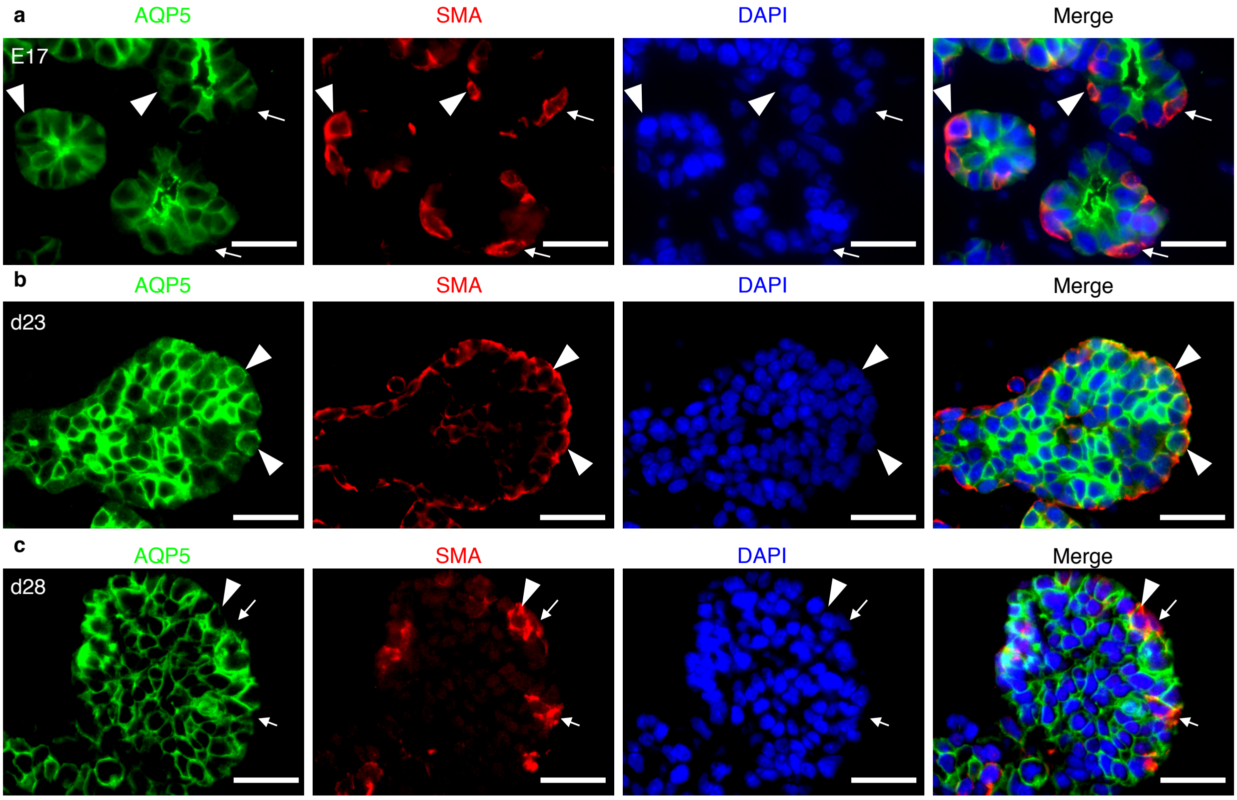

Supplementary Figure 6. End buds of embryonic salivary glands and iSGs co-express acinar and myoepithelial markers. **a** Immunofluorescence staining of AQP5 (green) and α-SMA (red) for embryonic salivary gland on E17. AQP5 and α-SMA double-positive polygonal cells observed in the end buds (arrowhead). α-SMA single positive spindle cells detected in the end buds (arrow). **b** The outer cells of iSG on d23 consisted of AQP5 and α-SMA double-positive cells (arrow). **c** The outer cells of iSG on d28 contained AQP5 and α-SMA double positive polygonal cells (arrowhead) and α-SMA single-positive spindle cells (arrow). Representative images from one out of more than 6 glands or iSGs are shown. The nucleus can be seen in blue (DAPI). Scale bars, 50 μm.

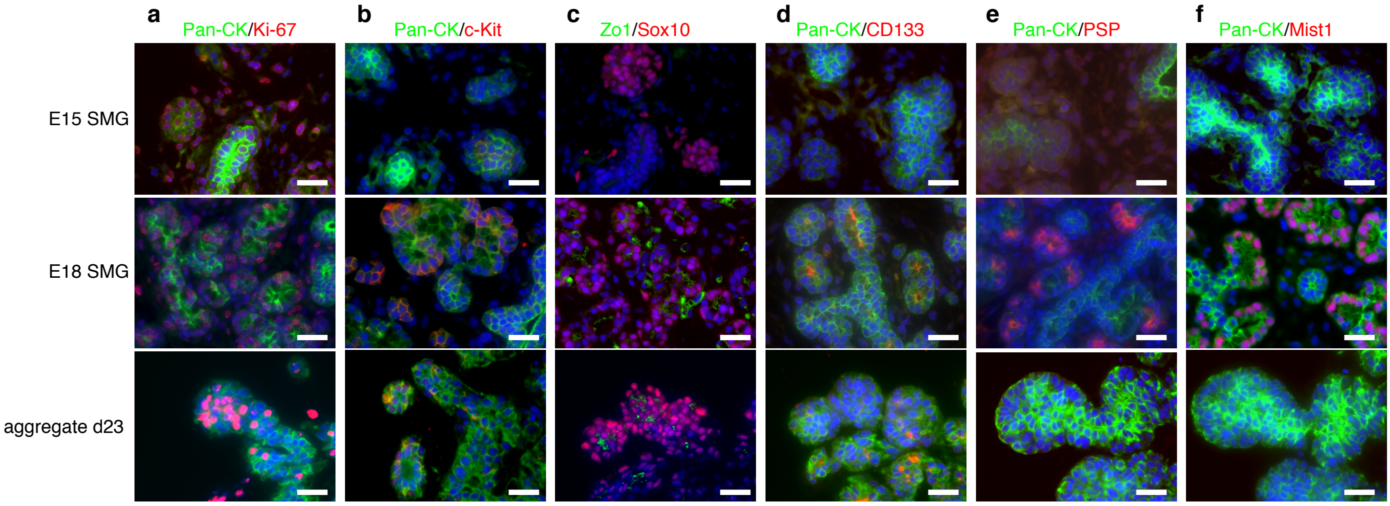

Supplementary Figure 7. Characterization of ESC-derived branching structure. **a-f** Immunofluorescence staining of E15 SMG, E18 SMG, and ESC-derived branching structures on d23. (a, b) Ki-67-expressing cells, which have proliferative ability, and c-Kit-expressing stem/progenitor cells, existed in epithelial cells of E15 SMG, E18 SMG, and ESC-derived branching structures on d23. (c) Sox10-positive cells were observed in end buds of E15 SMG, E18 SMG, and ESC-derived branching structures on d23. Zo-1-positive lumen structures were detected in E18 SMG, but not E15 SMG, and also observed in ESC-derived branching structures on d23. (d) CD133-positive lumen structures were detected in E18 SMG, but not E15 SMG, and also observed in ESC-derived branching structures on d23. (e, f) Mist1- and PSP-positive acinar cells were detected in E18 SMG, but not E15 SMG and ESC-derived branching structures on d23. The nucleus can be seen in blue (DAPI). Scale bars, 50 μm.

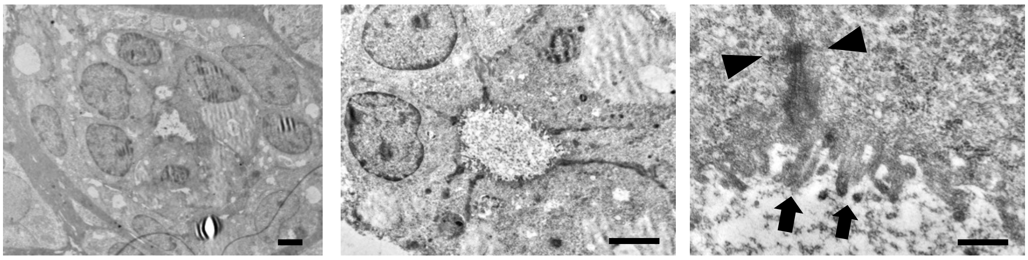

Supplementary Figure 8. Ultrastructure of the induced salivary gland. Transmission electron microscopy analysis of the ESC-derived branching morphology on d23 of differentiation. Acinar-like structures formed in the lumen and consisted of luminal cells and basal cells (left). Tight junctions (arrowhead) and microvilli (arrow) were observed on the luminal side (middle and right). Scale bars, 2 μm (left and middle), 500 nm (right). Representative images from 3 experiment are shown.

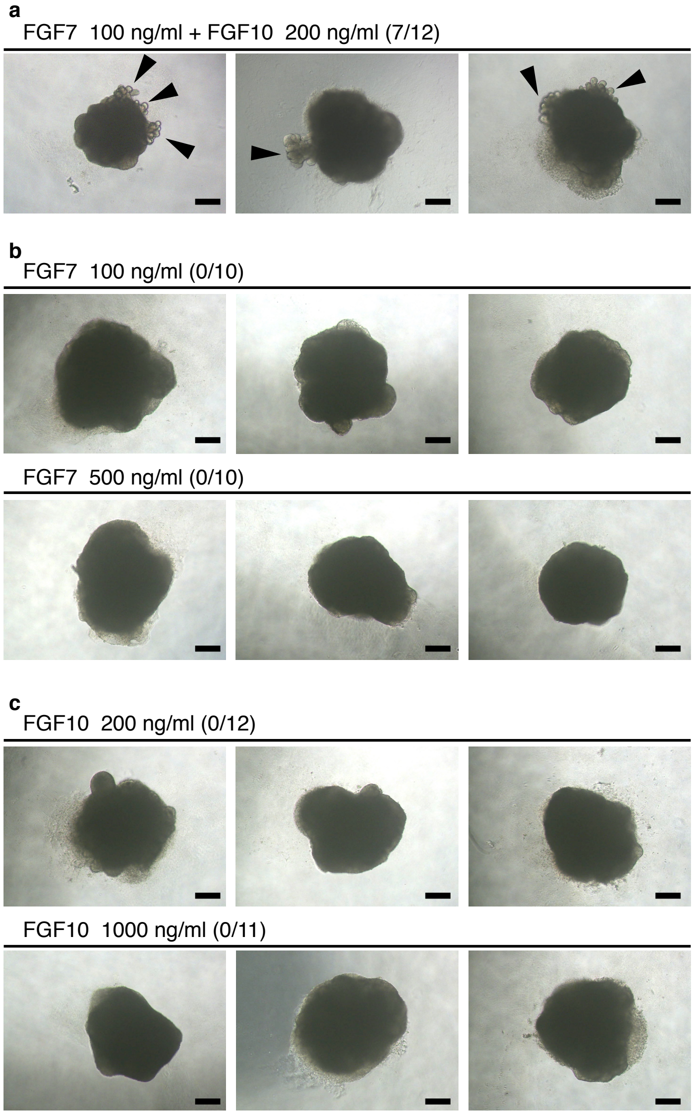

Supplementary Figure 9. Combination of FGF7 and FGF10 have distinct morphological effects on iSG culture. **a** Combinations of FGF7 and 10 induced embryonic salivary gland-like structures on d28. Single or multiple salivary gland-like branching structures observed in the aggregate. Efficiency of salivary gland-like structure formation are shown in parentheses. Arrowhead indicate salivary gland-like structures. **b** Salivary gland-like structures were not detected in two concentrations (100 ng/ml (top) and 500 ng/ml (bottom)) of FGF7 without FGF10 treated aggregates on d28. Salivary gland-like structures formation efficiency are shown. **c** Salivary gland-like structures were not detected in two concentrations (200 ng/ml (top) and 1000 ng/ml (bottom)) of FGF10 without FGF7 treated aggregates on d28. Efficiency of salivary gland-like structure formation are shown in parentheses. Representative images from more than 10 aggregates are shown. Scale bars: 300 μm.

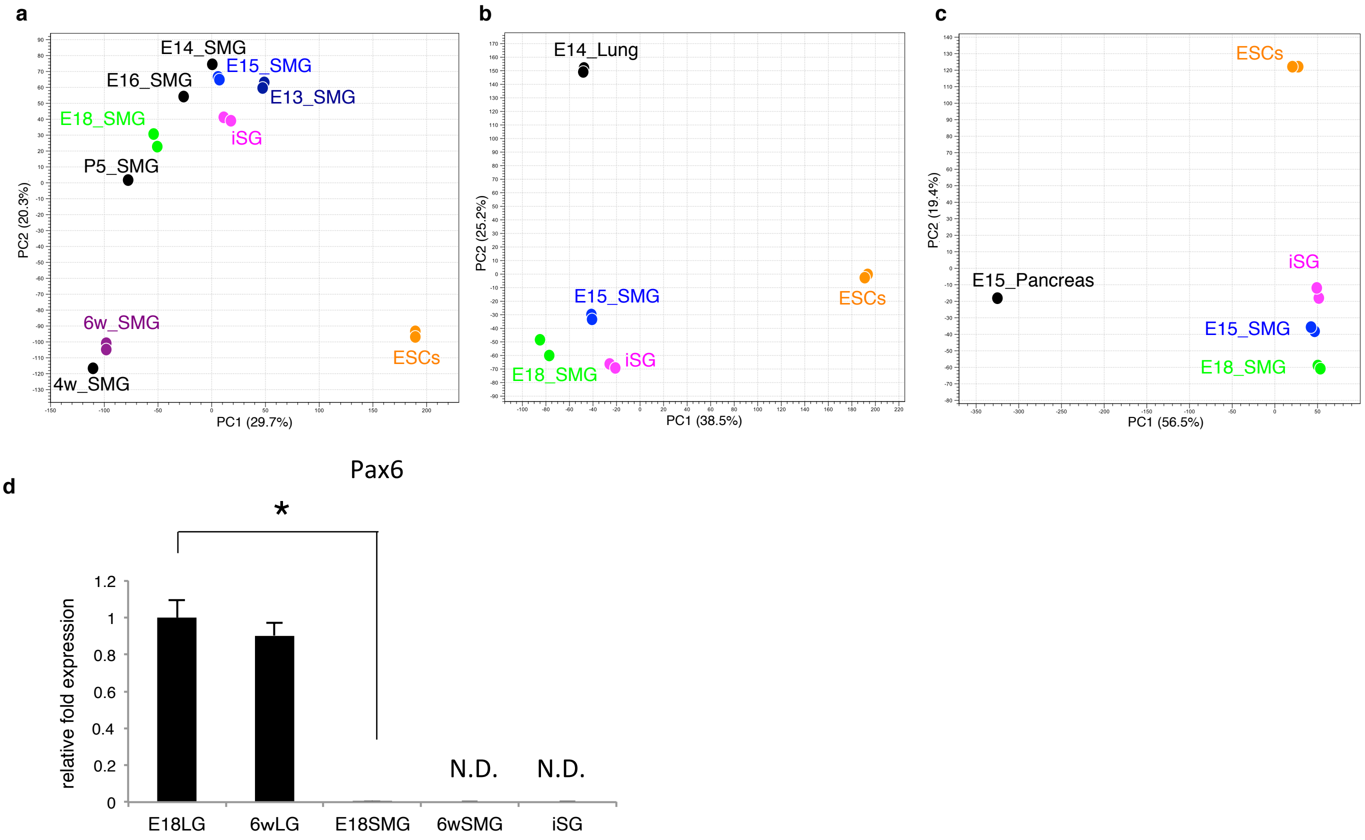

Supplementary Figure 10. Principal component analysis of the salivary glands at different developmental time points and other embryonic branching organs. **a** Results of a principle component analysis based on the global gene expression (examined by RNA-seq) comparing iSG on d23, ES cells (EB5), embryonic salivary glands on each stages in this study (E13, E15, E18, 6w) and deposit dataset (E14, E16, P5, 4w). **b** Results of a principle component analysis based on the global gene expression (examined by RNA-seq) comparing iSG on d23, ES cells (EB5), embryonic salivary glands on two stages (E15, E18), and the embryonic lung tissues on E14. There is a striking separation between iSGs and the embryonic lung tissues on E14. **c** Results of a principle component analysis based on the global gene expression (examined by RNA-seq) comparing iSG on d23, ES cells (EB5), embryonic salivary glands on two stages (E15, E18), and the embryonic pancreas tissues on E15. There is a striking separation between iSGs and the embryonic pancreas tissues on E14. **d** Pax6 (marker of lacrimal glands) expression levels in embryonic SMG, iSG on d23, embryonic lacrimal glands, and adult lacrimal glands were evaluated via real-time RT-PCR. The presented results are expressed as the mean ± S.D. Statistical analyses were performed using Student's t-test. **P* = 0.001. This experiment was replicated three times with similar results.

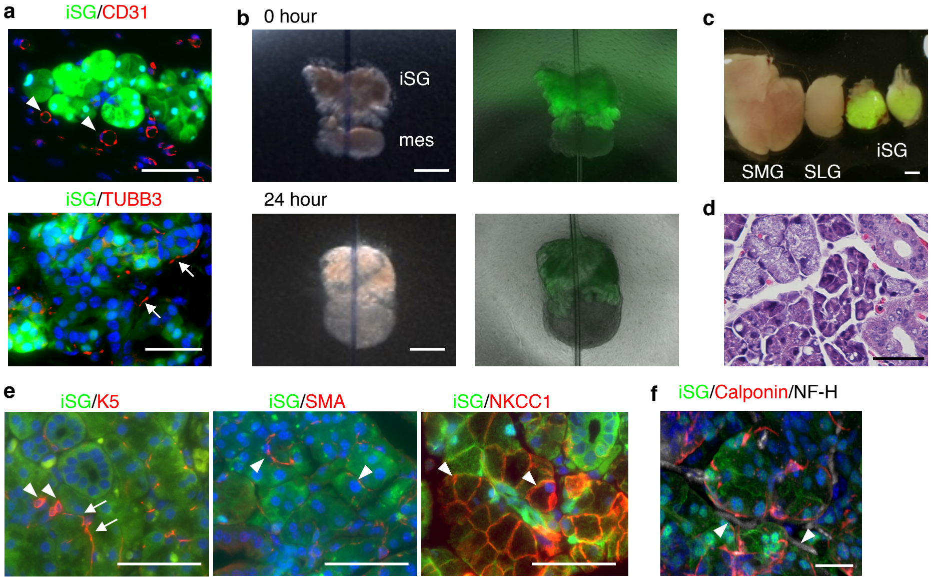

Supplementary Figure 11. Transplantation of induced salivary glands. **a** Immunofluorescence analysis of transplanted iSGs. CD31-positive vessels and TUBB3-positive nerve fibres were observed in GFP-negative mesenchyme around GFP-positive parenchyme. Both the vessels and nerve fibres were GFP-negative cells. Representative images from one of three transplanted iSGs are shown. The nucleus can be seen in blue (DAPI). Scale bars, 50 μm. **b** Representative phase-contrast images of a co-cultured GFP-labelled iSG with normal mouse-derived mesenchymal cells containing a PGA monofilament guide (left). Images show the initiation of co-culture (top) and after 24 hours (bottom). Phase-contrast images were merged with the corresponding GFP images, respectively (right), Scale bars, 300 μm. **c** Photographs of the normal 6-week-old mouse SMG, sublingual gland (SLG), and GFP-labelled iSGs transplanted with mesenchymal cells in salivary gland-defective mice at 30 days after transplantation. Representative images from one out of 10 transplanted iSGs are shown. Scale bar, 1 mm. **d** H&E staining of the iSG transplanted with mesenchymal cells. Scale bar, 50 μm. **e** Immunofluorescence analysis of the engrafted iSG transplanted with mesenchymal cells. Each image was merged with the corresponding GFP image. K5-positive signals were localized in the cytoplasm of basal (arrowhead) and myoepithelial cells (arrow). α-SMA-positive signals were localized in the cytoplasm of myoepithelial cells (arrowhead). NKCC1-positive signals were localized to the basolateral membrane of acinar cells (arrowhead). Representative images from one out of three transplanted iSGs are shown. The nucleus can be seen in blue (DAPI). Scale bars, 50 μm. **f** Fluorescent images are 4 μm projections of 50 μm sections. Merged images of GFP (green), calponin (red), and neurofilament H (NF-H) (white) are shown (right). Arrowheads indicate nerve fibers. The nucleus can be seen in blue (DAPI). Scale bars, 50 μm.

Supplementary Table 1. Up-regulated transcription factors in stalk and bud, compared with OE

Feature ID	stalk vs OE	bud vs OE
Ehf	28.7977418844275	15.964217170029
Sox10	10.0471142741452	63.0072899314807
Gata3	8.64525616599125	10.5173648806688
Cebpb	7.05741647242029	15.9999826924492
Foxc1	6.81101712527808	10.1941723511445
Tfap2b	6.60601583050301	28.6012247885285
Barx2	6.43101739656418	5.87245473590589
Elf3	5.60102234583901	3.32258795517564
Foxa1	5.41014257644058	8.6608179108595
Hey1	4.95722667390349	5.21996055063121
Sox9	3.92592412790539	7.14520353847394
Sox13	3.17706457895262	4.38548821386259
Dmrtb1	2.96146009090338	3.19434771830922
Tfap2a	2.9556074029767	4.22874830658718
Zbtb33	2.67306974897203	3.53964529389095
Eya2	2.63552604370872	2.49609298014518
Rbpms	2.61250417597022	4.55647481920949
Trnp1	2.56326054566919	9.71516945957791
Jdp2	2.19444331186623	3.06309891283595
Myc	2.17664250987723	4.65815685560773
Nrip1	2.04902839361003	2.64416479628753
Six1	2.00504956787857	3.75777990690434

Supplementary Table 2. Overlapping genes between up-regulated genes by RNA-seq and putative Sox9 target genes obtained in ChIP-seq in SMG

Peak rank	chr	start	end	gene_name	Distance to_TSS	Distance to_TES	location
39	chr9	71744079	71744261	Cgnl1	-124762	-269855	TSS_upstream
63	chr11	112572983	112573130	Sox9	-70481	-76006	TSS_upstream
86	chr6	93012078	93012220	Adamts9	-160715	-289457	TSS_upstream
87	chr11	113093440	113093592	Sox9	449979	444454	TES_downstream
94	chr10	98859014	98859167	Dusp6	133226	128973	TES_downstream
97	chr1	130546147	130546273	Cxcr4	-57335	-61435	TSS_upstream
123	chr2	170336745	170336863	Dok5	-220635	-368463	TSS_upstream
128	chr3	69120364	69120478	Kpna4	-189408	-244279	TSS_upstream
140	chr12	74549857	74549995	Tmem30b	97455	94174	TES_downstream
148	chr15	61450761	61450894	Myc	-366068	-371088	TSS_upstream
159	chr15	101114396	101114525	Nr4a1	17168	9238	TES_downstream
171	chr10	97755006	97755150	Atp2b1	-622725	-731343	TSS_upstream
183	chr12	70603123	70603264	Arf6	130057	126227	TES_downstream
187	chr6	127299895	127300074	Ccnd2	-198919	-224258	TSS_upstream
189	chr10	83650436	83650568	Appl2	-539094	-587724	TSS_upstream
193	chr1	43415158	43415284	Nck2	-87374	-212135	TSS_upstream
198	chr14	102053039	102053183	Uchl3	-72	-42230	TSS_upstream
206	chr15	56994073	56994177	Has2	-468025	-496944	TSS_upstream
208	chr15	63507102	63507254	Myc	1690283	1685263	TES_downstream
213	chr16	22455902	22456043	Etv5	-16330	-74587	TSS_upstream
249	chr9	60629613	60629793	Uaca	-12651	-98473	TSS_upstream
259	chr16	85128513	85128674	App	45354	-173906	intron
262	chr16	76866260	76866402	Nrip1	-493038	-575225	TSS_upstream
271	chr12	25221928	25222073	Taf1b	38504	-21228	intron
271	chr12	25221928	25222073	Grhl1	-43583	-80252	TSS_upstream
278	chr18	44913537	44913645	Mcc	-91672	-328878	TSS_upstream
278	chr18	44913537	44913645	Mcc	58244	-328878	intron

Peak rank	chr	start	end	gene_name	Distance to_TSS	Distance to_TES	location
280	chr14	59407155	59407318	Fgf9	715714	678226	TES_downstream
289	chr13	72335728	72335877	Irx1	-234632	-240123	TSS_upstream
289	chr13	72335728	72335877	Irx2	-430623	-435839	TSS_upstream
295	chr3	37399930	37400062	Spry1	-138901	-143522	TSS_upstream
296	chr2	165490749	165490843	Eya2	9999	-106334	intron
303	chr6	54700314	54700471	Znrf2	-66517	-139337	TSS_upstream
307	chr6	98922709	98922891	Foxp1	190211	-42717	intron
308	chr6	137645270	137645422	Eps8	-47706	-219581	TSS_upstream
308	chr6	137645270	137645422	Strap	-38256	-55104	TSS_upstream
311	chr8	90787142	90787254	Heatr3	125415	91357	TES_downstream
312	chr16	36771143	36771259	Slc15a2	13846	-20921	intron
313	chr17	14281198	14281358	Dact2	59822	51272	TES_downstream
318	chr5	73646044	73646194	Fryl	-86442	-155367	TSS_upstream
319	chr3	27920126	27920242	Fndc3b	-310886	-605102	TSS_upstream
328	chr15	95521780	95521898	Dbx2	-36638	-67846	TSS_upstream
329	chr1	135389106	135389231	Sox13	-68380	-110292	TSS_upstream
353	chr15	79023784	79023926	Polr2f	52059	41659	TES_downstream
354	chr15	55671958	55672094	Has2	854074	825155	TES_downstream
355	chr15	55256555	55256712	Col14a1	117328	-95722	intron
363	chr7	80536984	80537101	Rgma	16650	-27740	5'UTR
364	chr10	111275748	111275896	Phlda1	332481	330119	TES_downstream
375	chr8	86224811	86224904	Ddx39	-14240	-22389	TSS_upstream
378	chr2	79372901	79372928	Ssfa2	-102782	-138076	TSS_upstream
381	chr6	134279764	134279905	Etv6	294110	59670	TES_downstream
390	chr5	23122312	23122509	Pus7	167068	124103	TES_downstream
401	chr11	113138552	113138729	Sox9	495103	489578	TES_downstream
456	chr14	102298401	102298554	Uchl3	245294	203136	TES_downstream
461	chr15	32492569	32492711	Sema5a	318073	-133455	intron
470	chr16	76851676	76851817	Nrip1	-478453	-560640	TSS_upstream
474	chr12	95705418	95705559	Flrt2	-1224975	-1313746	TSS_upstream

Peak rank	chr	start	end	gene_name	Distance to_TSS	Distance to_TES	location
475	chr5	23122612	23122789	Pus7	166778	123813	TES_downstream
489	chr9	31495748	31495888	Barx2	225051	157810	TES_downstream

Supplementary Table 3. Branching formation of aggregates under different experimental conditions

FGF7	FGF10	Ad-βgal	Ad-Sox9	Ad-Foxc1	Branching/Aggregates [*n*]
+	+	-	+	+	11/25
+	+	+	-	-	0/16
+	+	-	+	-	0/20
+	+	-	-	+	0/16
-	-	-	+	+	0/18
-	-	+	-	-	0/20
-	-	-	+	-	0/20
-	-	-	-	+	0/20
+	-	-	+	+	0/10
-	+	-	+	+	0/12

Supplementary Table 4. Proteome analysis of salivary secreted protein

Protein name	Gene name	iSG	whole saliva
Vomeromodulin	Bpifb9a	183,780,216	23,190,970
Odorant-binding protein 1b	Obp1b	118,035,251	31,842,407
Odorant-binding protein 1a	Obp1a	50,703,043	13,782,697
Mucin-19	Muc19	711,011	33,593,574
Acidic mammalian chitinase	Chia	83,459	49,626,735
Carbonic anhydrase 6	Ca6	1,020,485	60,917,102
Cysteine-rich secretory protein 1	Crisp1	133,996	10,442,832
Kallikrein-1	Klk1	353,604	110,779,309
Alpha-amylase 1	Amy1	106,861	188,647,228
Prolactin-inducible protein homolog	Pip	15,518,444	92,148,601
