# Supplementary material for: Generation of orthotopically functional salivary gland from embryonic stem cells
Source: Nat Commun. 2018 Oct 11;9:4216. doi: 10.1038/s41467-018-06469-7 (PMC6181987; doi:10.1038/s41467-018-06469-7)
Supplement: Supplementary file 3 — Description of Additional Supplementary Files [file 41467_2018_6469_MOESM3_ESM.docx]

**Description of Supplementary Data and Supplementary Movies**

File Name: Supplementary Data 1

Description: RNA-Seq analysis of laser micro-dissected tissues of the SMGs at E12.5.

File Name: Supplementary Data 2

Description: Sox9 ChIP-seq analysis in SMGs at E13.5.

File Name: Supplementary Data 3

Description: Intersection of genes associated with Sox9 ChIP-seq peaks in salivary glands and those in pancreatic progenitors.

File Name: Supplementary Data 4

Description: RNA-seq analysis of iSGs at d23, transplanted iSGs, transplanted combined iSGs, ESCs, and embryonic SMGs in each developmental stage.

File Name: Supplementary Data 5

Description: Proteome analysis of saliva secreted from iSGs.

File Name: Supplementary Data 6

Description: Gene expression of oral epithelium on iSG saliva that are not present in whole saliva.

File Name: Supplementary Movie 1

Description: Fluorescence image of intracellular Ca2^+^ release in iSGs induced by 100 μM Charbacol. Changes of Fluo-4 fluorescence were displayed.

File Name: Supplementary Movie 2

Description: Fluorescence image of intracellular Ca2^+^ release in iSGs induced by 10 μM Charbacol. Changes of Fluo-4 fluorescence were displayed.

File Name: Supplementary Movie 3

Description: Fluorescence image of intracellular Ca2^+^ release in iSGs induced by 100 μM Charbacol. iSGs were pretreated with atropine. Changes of Fluo-4 fluorescence were displayed.
